# Achieving the Health Equity Agenda Through Transformative Community-Engaged Strategies

**DOI:** 10.5888/pcd20.230077

**Published:** 2023-11-09

**Authors:** Keon L. Gilbert, Mary Shaw, Arjumand Siddiqi, Melody S. Goodman

**Affiliations:** 1 Saint Louis University, Department of Behavioral Science and Health Education, Saint Louis, Missouri; 2 Brookings Institution, Washington, DC; 3 Jackson State University, Department of Behavioral & Environmental Health, College of Health Sciences, Jackson, Mississippi; 4 University of Toronto, Dalla Lana School of Public Health, Hospital for Sick Children, Toronto, Ontario, Canada; 5 New York University, School of Global Public Health, New York, New York

Achieving health equity is a realistic vision. However, the process, goals, metrics, and strategies needed to provide access to resources for all citizens are not truly aligned with American values and traditions (1). The current vision for health equity is being neutralized because racism is the root of health inequalities and is deeply embedded in our approaches to solving health challenges (2,3). One of the primary strategies public health has developed is to engage communities experiencing disproportionate rates of illness and lower life expectancy. The main question for us to wrestle with is, has community engagement realized its full potential to move the field toward achieving health equity? This question has been relevant since the adoption of community engagement strategies, as they have been framed in public health as community-based participatory research (CBPR) approaches. The field of public health adopted CBPR nearly 3 decades ago as an alternative paradigm to understand the complexity of health challenges and to develop strategies for health promotion change that explicitly included communities as equal decision makers. The COVID-19 pandemic documented the challenges of community relationships between public health agencies, research institutions, and health care facilities. Communities that are disengaged from decision making about their health lack trust and respect for these organizations charged with preventing disease and enhancing health. To move into a new generation of health promotion and health equity, we pose 2 questions and provide some direction toward answering them: 1) What more can CBPR do to become a leading paradigm to help achieve equity in health outcomes? and 2) How can CBPR prevent itself from becoming a gatekeeper to social and structural change?

## The Health Equity Challenge

For the past several decades, health disparities have been a central part of the discourse in public health circles (4). Research on health disparities and social determinants of health has proliferated over time and has largely focused on documenting existing disparities and noting some promising interventions that include health equity and integrating practices of anti-racism (4–6). One of the most interesting elements of these efforts has been strategies to bridge the distance between researchers trained to produce rigorous evidence and the communities intended to benefit from this research. Communities that are the object of these rigorous approaches remain absent from informing the research process, intervention development, policy change, interpreting results, and developing community-tailored strategies to be adopted. Without community-engaged and community-led research, disparities and inequalities will persist and widen. For example, Chetty and colleagues found that life expectancy disparities across income quartiles widened between 2000 and 2014 (7). This increase in disparities appeared to have been driven by larger gains in life expectancy accruing to the top income quartile compared with the bottom income quartile, rather than declines in life expectancy in the bottom income quartile. In another example, racial disparities in infant mortality were constant during the 2000s (driven by similar declines in infant mortality in both Black and White infants, but not enough decline in Black infants to reduce the gap); however, by the 2010s, infant mortality for Black infants stopped declining but continued to decline among White infants, therefore exacerbating these disparities. Not solving health inequalities is an act and byproduct of systematic denial and systemic racism. Our inability to solve persistent health challenges for vulnerable populations reproduces inequality across family and community generations. CBPR has been used as an approach in public health to address systemic change through initiatives and methods that build on working with communities to enhance the capacity to create sustainable change strategies (8–11).

## The Rise of Community-Based Approaches to Solve Health Inequalities

As CBPR was being defined and established as a new paradigm in public health, social determinants of health (SDOH) were being translated from other social science disciplines to frame the root or upstream causes of health behaviors and access to health care and social services (12). SDOH gave new language to the field to analyze causal influences based on social structures, historical injustices and trauma, and policy decisions that create inequitable access to social services, educational opportunities, employment, and quality housing (13–15). Our field has also addressed the policy making process within government to take on a health analysis or for these policies to address potential health effects (16). Most recently, systemic racism has been acknowledged by mainstream public health entities as a fundamental determinant of health (4,17–20). These developments in the field are necessary ingredients to invite different orientations to research, practice, and policy making that remove the false divide between research and practice (3,21). CBPR has become a model to shift power relationships between researchers and communities affected by health inequalities.

The central focus of CBPR is to find equity in collaborations by joining the strengths of researchers and communities to eliminate health disparities. As a field, public health has invested resources such as contracts and grants, developed training programs, and developed other curricula within schools and programs of public health to justify CBPR as a legitimate research paradigm purporting that community engagement achieves long-term strategies to address historic, chronic, and acute health issues, by eliminating their structural barriers. A major focus of CBPR and other participatory approaches is the process of inclusion (22), with the intention that disparities can be eliminated. An approach that integrates research, action, and education has helped to reimagine many of our existing study designs, methods, and intervention approaches. Integration approaches are not enough to address historic, chronic, and persistent socioeconomic and political disenfranchisement. A transformative approach is needed to enable social and structural changes at the base of Frieden’s health pyramid (23). This work also draws on the theoretical and practice roots espoused by Paulo Freire by embracing a critical consciousness or research paradigm that spurs a cycle of critical introspection and action. We no longer need an integrationist approach to CBPR. It has been established in the field of public health and within the academy for more than 3 decades. An integrationist approach created the pathways for scholars to engage in research using academic–community partnerships. This reoriented the public health infrastructure to create new support mechanisms oriented toward equitable decision making (24).

A transformative approach creates pathways to build more centers of excellence to engage in CBPR and to reorient public and private institutions toward building stronger social bonds with marginalized communities to enhance health outcomes. To build these centers of excellence and improve partnership capacities, we need transformative approaches that are more closely aligned to the social infrastructure and social capital ties within marginalized communities. This type of strategy reduces barriers to partnership development and builds trust because the research is informed by community members’ voices, and interventions and programs reflect that community’s assets and strategies for resistance, resilience, and community building (25–27).

CBPR offered public health a different approach to research by integrating marginalized voices. Centering marginalized voices is possible by following the development and progress of feminist and womanist participatory approaches, intersectional theory and research, and the adoption of critical race theory into public health (28–30). Feminist and womanist participatory approaches begin by deconstructing the Eurocentric bias inherent in traditional research methods (28,29,31). Eurocentric research approaches impose a methodologic hierarchy, which is counter to the lived experiences of marginalized groups and interferes with research that can be iterative and emergent (32). Our field’s reliance on “gold standard” methods has created occupational hazards for scholars of color and other underrepresented scholars to engage in academic research because they are forced to use these methods for advancement. Structural, anti-racist change is needed in the field of public health that will support underrepresented scholars and their goals to engage in non-Eurocentric research. Feminist or womanist and intersectional researchers, especially those led by Black women scholars, pushed the academic boundaries for inclusion as a field of study, ensured that scholarship about Black women included Black women, and used methods that centered the intersections of Black women. Participatory approaches hold the same capacity. Participatory approaches include principles and processes to a) develop study designs agreed upon as a collective of equals, and b) select the method that best fits the population of focus, the public health problem, and the solution.

The second step allows CBPR to be shaped by the social identities of marginalized people or groups experiencing higher rates of illness, death, and disability. In practice, the convergence of social identities is not ignored or isolated, nor are the identities treated solely as variables (33). The practice of centering marginalized voices moves beyond simply hearing their experiences of discriminatory behaviors in social and health care systems to creating within- and across-system changes to prevent future experiences.

Bowleg takes this idea of social identity further by challenging federal policy and research institutions to become more inclusive in their language about women and minorities (34). The documentation of categories of social identities has been inadequate in documenting the impact of interlocking systems of privilege and oppression. How this translates for researchers and practitioners is to encourage them to focus their work on the intersections of social identities and to no longer investigate these identities individually. This approach underscores Chetty’s findings and explains the multiple generations of Black women with varying levels of socioeconomic status (SES) who remain at a higher risk for maternal and infant mortality, as well as the Black men with varying levels of SES who experience a higher risk of being victims of police killings and who experience lower levels of intergenerational wealth transfers. For Black women and challenges related to Black maternal and child health, previous research has sought to address individual, family, and health care risks and factors related to neighborhoods and SES. Black women scholars and practitioners reclaimed the narratives about Black women and created tailored strategies for Black birthing people. Black women scholars, practitioners, physicians, community health workers, doulas, and activists took the lead in identifying intersectional approaches to reduce this growing health inequality (35–37). These efforts highlight the role of intersecting social identities and the praxis of knowing that single-social-identity–based research cannot fully explain (quantitatively, qualitatively, or ethnographically) the totality of inequality or opportunity.

A third step advances participatory research that includes intersectional research of social identities, which will reframe our current CBPR models that uplift the role of context, place, or both in research and interventions. Traditional research approaches use context as settings or locations to recruit research and intervention participants. Examples include schools, churches, clinics, community centers, and health departments. Participatory models rely on community members to develop research and intervention designs that integrate values, social norms, traditions, and practices of each unique community or population context. However, more work is needed during this era of anti-racism and public health to more fully integrate, examine, and create change within the social structures and settings to focus on health equity.

Previous work in social settings owned, operated, managed, and governed by policies and practices of white supremacy have had a diminished focus on structural change, anti-racism, and health equity. Within marginalized communities, settings such as Black neighborhoods, Black-owned businesses, Black faith institutions) are safe havens for community organizing and mobilization (38,39). CBPR partnerships have focused on building the capacity of community-based organizations. Some work has been done to change health care settings; one example of anti-racism in practice is to change health systems and health outcomes (40). Our public health interventions have evolved to be uniquely tailored to certain subpopulations or specific settings. Still, they lack a larger population focus, which fosters changes across social stratification and includes system changes that meet the needs of intersecting social identities (21,41). Any research or intervention that does not include a focus on health equity and anti-racism strategies within the organization and explicitly focus on changing social and health outcomes will have a limited impact on population-level outcomes. Without an intentional goal of changing social structures to achieve health equity using anti-racism strategies, we will render our public health efforts null and will reflect status quo research. Much of our public health efforts are limited to changing interpersonal behaviors, such as better communication between providers and patients, or focused only on improving individual health behaviors, such as healthy eating and physical activity. These successes, however, relegate our public health efforts to proximal changes without sustainable social or structural change.

## Social Movements Inform Participatory Approaches

External social movements have shaped many advances in academic research. These social movements highlight the human condition in a way that traditional research cannot. Marrying participatory methods to the goals of social movements catalyzes the transformational power of CBPR. This links the intersections of social identities and lived experiences and should create structural change in our research approaches. Recently, academic research has focused on racism as a public health crisis fueled by the Black Lives Matter movement. This movement fundamentally calls for ending anti-Black racism across all public and private sectors. The end of anti-Black racism does not suggest it is more important or unrelated to the end of other forms of systemic oppression. It raises the social identities of Black people as the focus of systemic discrimination and as the focus of any social and policy-based remedies. During the anti-slavery abolitionist movement, this was captured by divisions between Black and White abolitionists who agreed with the inhumanity of the institution of chattel slavery. However, these abolitionists disagreed about the human, social, and political rights Black people should have to be full citizens. In another example, borrowing from the criminal justice reform movement, many formerly incarcerated individuals cannot access their full civil and political rights because of laws that politically disenfranchise them (42,43). Many citizens cannot access social services or public assistance benefits because of felony or misdemeanor convictions. These examples show that we stratify access to human, civil, and political rights based on social identities. Therefore, our solutions to these differential access points must include the voices of those affected, embodying their social identities and realities. These examples highlight the role of understanding how social identities may be relegated to permanent status without social, structural, or policy change to create more fluidity and equitable access to resources.

## Roles CBPR Approaches Can Adopt to Become Transformational

*The role and social identities of both the researcher and communities involved*. In a recent article, Wright et al take on this perspective as Black women scholars in public health. They frame their identities as “outsiders within” to confront racism within the academy and the challenges they encountered to lead a new approach to their research. They challenged the academy to recognize their social identities and human experience as Black women, scholars, and mothers (37). They described how their social identities help them frame the design of their research, collect data, and work with communities to interpret these data from a Black woman–centered lens. Their capacity to work with marginalized communities mirrors their own critical consciousness to create academic pathways to engage in participatory research with strategies of anti-racism to achieve health equity.

*The roles and identities of public, private, and community-based organizations.* Institutional and organizational change is often difficult to achieve and requires internal capacity building in response to external factors. We recognize the field of public health has become energized to focus on health equity. However, our public health institutions and settings need to develop the capacity to adopt a similar vision, which will lead to abolishing policies and practices across the public health infrastructure that obstruct participatory approaches and resist adopting strategies for anti-racism. This calls for reorienting public, private, and community-based organizations to focus on equity in health for specific populations in partnership to address the broader social structures. This ultimately means the social structure within each organization has to morph by undoing racism within. If more anti-racism work is adopted and implemented, it can reframe the core of our public health system, which was not built to address health disparities or health inequities.

*Examining and upending racial capitalism in public health*. An examination of the racial foundations of capitalism within a US context has the potential to open a complex analysis of our public health infrastructure to allow it to become better oriented toward health equity. The national focus on health disparities since the Heckler Report of 1985 has created a health disparities industry (44); CBPR has created a parallel industry. Focusing solely on creating equity within partnership structures without an equal focus and supporting resources on systemic change within each structure and partnering organization may relegate CBPR to a powerless tool to achieve health equity. The future of CBPR should work toward a vision of health equity and avoid becoming an industry of reproducing health inequalities and status quo research without creating structures for systemic and social change. Our political structures, funding models, and policies of academic institutions are conservative at best and lack transformative power (24,45). Racial capitalism exists within public health and its approaches because of its ties to funding practices that limit the work to individual change and biomedical models (46,47).

## Conclusion

Collective action in the US and globally is not a new form of mobilizing individuals, groups, and communities to advocate for structural change. Over the last 2 decades, the US has experienced a rise in collective action to directly challenge income inequality, police killings, educational gaps, environmental racism, and, most recently, removing federal protections for reproductive choices. CBPR, as a strategy based on collective action, community organizing, and mobilization, has the potential to re-center public health’s focus on population-level change strategies. Twenty years after the landmark report, *Unequal Treatment* (48), our health care and other social service systems remain unchanged, leaving the US last in recent world rankings of health and health care (49). CBPR is one model for bringing researchers and communities together for equitable decision making about public health solutions. CBPR’s transformational role in public health can be an equal partner in creating structural changes within the academy, the broader public health workforce, and supporting community-based organizations’ capacity building, which can lead to systemic change across social, structural, and political determinants of health.
